# Immune Evasion in Head and Neck Squamous Cell Carcinoma: Roles of Cancer-Associated Fibroblasts, Immune Checkpoints, and *TP53* Mutations in the Tumor Microenvironment

**DOI:** 10.3390/cancers17152590

**Published:** 2025-08-07

**Authors:** Chung-Che Tsai, Yi-Chiung Hsu, Tin-Yi Chu, Po-Chih Hsu, Chan-Yen Kuo

**Affiliations:** 1 Department of Research, Taipei Tzu Chi Hospital, The Buddhist Tzu Chi Medical Foundation, New Taipei City 231, Taiwan; chungche.tsai@gmail.com (C.-C.T.); tintin4125@gmail.com (T.-Y.C.); 2 Department of Biomedical Sciences and Engineering, National Central University, Taoyuan 320, Taiwan; syic@ncu.edu.tw; 3 Department of Dentistry, Taipei Tzu Chi Hospital, The Buddhist Tzu Chi Medical Foundation, New Taipei City 231, Taiwan; 4 Institute of Oral Medicine and Materials, College of Medicine, Tzu Chi University, Hualien 970, Taiwan

**Keywords:** head and neck squamous cell carcinoma, tumor microenvironment, immune evasion, cancer-associated fibroblasts, regulatory T cells, myeloid-derived suppressor cells, PD-1/PD-L1, CTLA-4, natural killer cells, immunotherapy

## Abstract

Head and neck squamous cell carcinoma (HNSCC) is a highly aggressive malignancy characterized by complex interactions within the tumor microenvironment (TME) that facilitate immune evasion and tumor progression. The TME consists of diverse cellular components, including cancer-associated fibroblasts, immune and endothelial cells, and extracellular matrix elements, that collectively modulate tumor growth, metastasis, and resistance to therapy. Immune evasion in HNSCC is orchestrated through multiple mechanisms, including the suppression of cytotoxic T lymphocytes, recruitment of immunosuppressive cells, such as regulatory T and myeloid-derived suppressor cells, and upregulation of immune checkpoint molecules (e.g., PD-1/PD-L1 and CTLA-4). Natural killer (NK) cells, which play a crucial role in anti-tumor immunity, are often dysfunctional within the HNSCC TME due to inhibitory signaling and metabolic constraints. Additionally, endothelial cells contribute to tumor angiogenesis and immune suppression, further exacerbating disease progression. Recent advancements in immunotherapy, particularly immune checkpoint inhibitors and NK cell-based strategies, have shown promise in restoring anti-tumor immunity. Moreover, *TP53* mutations, frequently observed in HNSCC, influence tumor behavior and therapeutic responses, highlighting the need for personalized treatment approaches. This review provides a comprehensive analysis of the molecular and cellular mechanisms governing immune evasion in HNSCC with a focus on novel therapeutic strategies aimed at improving patient outcomes.

## Simple Summary

Head and neck squamous cell carcinoma is a challenging cancer that often resists treatment due to its ability to escape the body’s immune defense. This review explains how the tumor’s surrounding environment—made up of immune cells, blood vessels, and structural proteins—helps the cancer grow, spread, and avoid immune attack. We aim to highlight how different components in this environment, including fibroblasts, suppressive immune cells, and genetic changes like *TP53* mutations, contribute to disease progression. By understanding these mechanisms, researchers can develop more effective therapies that target not just the cancer cells, but also the supportive environment around them. This review may guide future research in creating personalized treatments and improving outcomes for patients with this aggressive cancer.

## 1. Introduction

Head and neck squamous cell carcinoma (HNSCC) is one of the most common malignancies, comprising a heterogeneous and aggressive group of tumors that arise from the mucosal epithelium of the oral cavity, pharynx (including nasopharynx, oropharynx, and hypopharynx), and larynx [1]. HNSCC is the sixth most common cancer worldwide, with over 800,000 new cases and more than 400,000 deaths annually, and is associated with significant morbidity and mortality [1]. Despite advances in surgical techniques, radiation therapy, and chemotherapy, the prognosis for patients with HNSCC remains poor, particularly in advanced stages [2]. This poor prognosis is largely attributed to the ability of HNSCC to evade immune surveillance, a process closely linked to the tumor microenvironment (TME) [3].

Major etiological factors contributing to HNSCC development include chronic exposure to tobacco-derived carcinogens and excessive alcohol consumption, both of which exert synergistic effects in promoting mucosal damage, genomic instability, and carcinogenesis. Infection with high-risk human papillomavirus (HPV), particularly HPV-16, has emerged as a distinct pathogenic driver of oropharyngeal squamous cell carcinoma [1,4]. HPV-positive HNSCC exhibits unique biological characteristics, including enhanced radiosensitivity, improved prognosis, and a distinct immune microenvironment compared to HPV-negative counterparts [5]. Additionally, Epstein–Barr virus infection, particularly in endemic regions, is a major risk factor for nasopharyngeal carcinoma, a subset of HNSCC [1].

Precancerous lesions such as oral leukoplakia, erythroplakia, oral submucous fibrosis, and epithelial dysplasia are well-established precursors of HNSCC and warrant careful surveillance and management [6,7,8,9]. Clinically, HNSCC typically presents as a painless mass, ulcer, or mucosal lesion, with symptoms such as dysphagia, odynophagia, hoarseness, or cervical lymphadenopathy depending on tumor location [10]. Diagnosis is based on histopathological examination revealing malignant squamous cells with varying degrees of differentiation, keratinization, and intercellular bridges [10].

Pathologically, HNSCC is graded by differentiation (well, moderately, or poorly differentiated) and staged using the TNM system, evaluating tumor extent (T), nodal involvement (N), and distant metastasis (M) [11,12]. Additional adverse pathological features include depth and pattern of invasion, perineural invasion, lymphovascular invasion, surgical margin status, and extracapsular extension of nodal metastases, all of which are associated with recurrence and poor prognosis [13].

Biomarker testing, such as p16 immunohistochemistry (IHC) as a surrogate for HPV infection in oropharyngeal tumors and programmed death-ligand 1 (PD-L1) expression assessment for immune checkpoint therapy eligibility, is recommended in clinical evaluation [14,15]. Most patients present with advanced-stage disease, and regional lymph node involvement is common, significantly impacting prognosis [12]. The clinical course is further characterized by locoregional recurrence and the emergence of second primary tumors due to field cancerization [16]. The overall 5-year survival rate remains approximately 50–60%, varying with anatomical site, HPV status, stage at diagnosis, and treatment response [17].

At the molecular level, HNSCC is marked by frequent mutations in tumor suppressor genes such as tumor protein p53 (*TP53*), cyclin-dependent kinase inhibitor 2A, and neurogenic locus notch homolog protein 1, as well as amplifications in oncogenes including cyclin D1 and epidermal growth factor receptor [18,19]. These genetic alterations contribute to dysregulated cell cycle progression, proliferation, and resistance to apoptosis.

Furthermore, HNSCC tumors exploit various immune evasion mechanisms, such as upregulation of immune checkpoint molecules (e.g., PD-L1 and cytotoxic T-lymphocyte-associated protein 4, CTLA-4), recruitment of immunosuppressive cells (e.g., regulatory T cells and myeloid-derived suppressor cells), and downregulation of major histocompatibility complex (MHC) class I molecules to escape cytotoxic T cell-mediated recognition [4,20].

Recent advances in immunotherapy, particularly immune checkpoint inhibitors targeting the PD-1/PD-L1 axis, have shown promising but limited efficacy in recurrent or metastatic HNSCC, underscoring the need for improved patient stratification and combination strategies [21,22]. Current research efforts are focused on integrating molecular profiling, TME characterization, and biomarker-guided treatment approaches to improve early detection, personalize therapy, and enhance clinical outcomes.

Understanding the interplay between etiological factors and tumor biology is crucial for developing effective prevention, early detection, and treatment strategies.

## 2. Tumor Microenvironment

**Figure 1:**
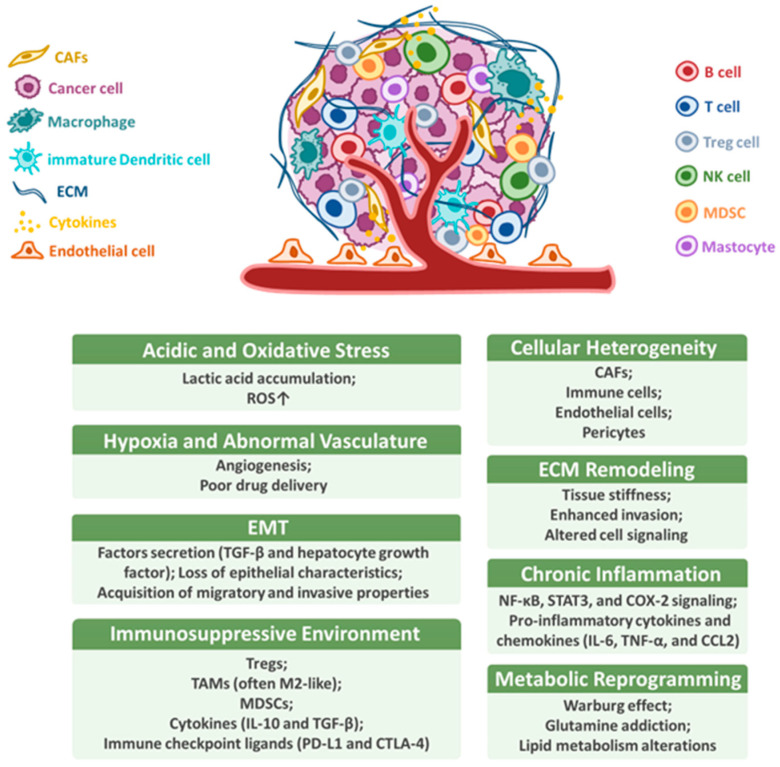
Characteristics of the tumor microenvironment. The TME comprises diverse cellular and non-cellular elements that promote immune evasion, tumor progression, and therapy resistance. Hallmark features include cellular heterogeneity (e.g., CAFs, Tregs, TAMs, MDSCs, and endo thelial cells), ECM remodeling, hypoxia and abnormal vasculature, immunosuppressive signaling (e.g., TGF-β, IL-10, and PD-L1), chronic inflammation, metabolic reprogramming (e.g., glycolysis and lactate), acidic and oxidative stress, and EMT. These interconnected features collectively define the aggressive and immune-evasive nature of HNSCC.

The TME is a complex and dynamic network of various cell types, extracellular matrix (ECM) components, and soluble factors that surround and interact with tumor cells [23]. In HNSCC, the TME plays a crucial role in promoting tumor growth, metastasis, and resistance to therapy [24]. Various elevated protein levels in tumor cells have been detected using IHC or immunofluorescence, such as PD-L1 and major histocompatibility complex (MHC) class I molecules [25]. These proteins reflect interactions between tumor cells and other components of the TME. Key components of the TME include cancer-associated fibroblasts (CAFs), immune cells, endothelial cells, and ECM proteins [26]. As shown in Figure 1, the TME is characterized by several interrelated features, including cellular heterogeneity, ECM remodeling, hypoxia and abnormal vasculature, immunosuppressive environment, chronic inflammation, metabolic reprogramming, acidic and oxidative stress, and the induction of epithelial–mesenchymal transition (EMT) [27,28]. These characteristics contribute to immune evasion, promote tumor progression, and hinder the efficacy of conventional therapies. The immunosuppressive milieu is enriched with regulatory T cells (Tregs), tumor-associated macrophages (TAMs), and myeloid-derived suppressor cells (MDSCs), which suppress cytotoxic T cell activity. Moreover, ECM stiffening and remodeling disrupt normal tissue architecture, while hypoxic conditions and aberrant angiogenesis further impair immune infiltration and drug delivery.

CAFs represent a major component of the TME and play a pivotal role in supporting tumor growth, invasion, and immune evasion [29]. Unlike normal fibroblasts, which are transiently activated during wound healing and return to a quiescent state upon tissue repair, CAFs exist in a persistently activated state within the TME [30]. This chronic activation is characterized by sustained expression of α-smooth muscle actin, fibroblast activation protein, and secretion of a wide array of pro-tumorigenic cytokines, growth factors, and ECM components [31]. The persistent activation of CAFs is driven by tumor-derived signals and maintains a feedforward loop that promotes cancer cell proliferation, angiogenesis, and immune suppression [32]. In HNSCC, CAFs have been shown to facilitate immune evasion by recruiting Tregs and MDSCs, and by modulating the expression of immune checkpoint molecules such as PD-L1 [33]. Understanding the stable activated phenotype of CAFs is critical for developing strategies to therapeutically target the tumor stroma and improve immune responsiveness.

CAFs persist in a chronically activated state within the TME, and are critical orchestrators of tumor progression and immune suppression in HNSCC. One of their principal functions is the secretion of a wide range of cytokines and growth factors that modulate immune cell behavior and facilitate tumor growth [34]. Notably, CAFs produce high levels of interleukin (IL)-6, a pro-inflammatory cytokine that promotes the expansion of MDSCs, enhances signal transducer and activator of transcription (STAT) 3 signaling in tumor cells, and impairs cytotoxic T lymphocyte activity [35]. Additionally, transforming growth factor (TGF)-β secreted by CAFs plays a dual role by promoting EMT in cancer cells [36]. Zhao et al. reported that a cluster of differentiation (CD) 68+ CAFs increased from dysplasia to oral squamous cell carcinoma (OSCC), and their presence in the tumor center correlated with better patient prognosis [37]. An interesting finding demonstrated that C-X-C motif chemokine ligand (CXCL)1/C-X-C chemokine receptor (CXCR)2 signaling is a critical pathway driving CAF differentiation and represents a potential therapeutic target in OSCC [38]. A previous study indicated that CAFs in OSCC secrete CXCL12, which attracts monocytes and promotes their differentiation into tumor-associated M2 macrophages via the CXCL12/CXCR4 pathway [39]. By shaping a microenvironment hostile to immune surveillance, CAFs act as key mediators of immune evasion in HNSCC, and targeting their signaling pathways may represent a promising strategy for improving immuno-therapeutic efficacy.

### 2.1. Role of CAFs and Immune Cells in the TME of HNSCC

CAFs are major cellular components of the TME in HNSCC [40]. These fibroblasts are activated by tumor cells and, in turn, secrete growth factors, cytokines, and ECM components that promote tumor proliferation, invasion, and angiogenesis [29]. Cytokines released by CAFs also influence the polarization and recruitment of immune cells [41]. The immune cell composition in the TME of HNSCC is heterogeneous and includes various populations of lymphocytes, macrophages, dendritic cells, and MDSCs [1,42]. Several studies have reported that HPV-associated tumors are primarily distinguished by a high abundance of tumor-infiltrating lymphocytes (TILs) [43,44]; however, myeloid cells are predominant in the TME of HPV-negative tumors [42,45]. Mandal et al. reported on the immune landscape of HPV-positive and HPV-negative HNSCC and provided a novel rationale for investigating agents that target modulators of Tregs, including CTLA-4, glucocorticoid-induced tumor necrosis factor receptor, inducible costimulatory molecule, indoleamine 2,3-dioxygenase, vascular endothelial growth factor A, and natural killer (NK) cells such as killer-cell immunoglobulin-like receptors, T cell immunoreceptors with immunoglobin and ITIM domains (TIGIT), and CD137 as adjuncts to anti-programmed cell death protein 1 (PD-1) in the treatment of advanced HNSCC [46].

TILs, particularly CD8+ cytotoxic T cells, are critical for anti-tumor immunity [47]. However, their function is often impaired in HNSCC due to the presence of immunosuppressive cells, such as Tregs and MDSCs [48]. Additionally, TAMs in HNSCC often exhibit a pro-tumorigenic M2 phenotype, which supports tumor growth and suppresses effective immune responses [49,50]. Troiano et al. demonstrated that the overexpression of M2-like CD163+ TAMs in patients with HNSCC is associated with poor clinical prognosis in terms of both overall survival (OS) and progression-free survival (PFS) [51], and a previous study indicated that CD163+ TAMs can serve as prognostic indicators in OSCC [52]. Moreover, improved relapse-free survival was associated with CD4+:CD8+ T cell ratios and CD39+CD73+CD19+ B cell proportions below the respective cohort medians [53]. Indeed, TILs show increased expression of CD8+, forkhead box protein P3 (FOXP3), and PD-L1 in the OSCC microenvironment, as demonstrated by IHC [25]. Interestingly, Moskophidis et al. found that T cell exhaustion is a condition of dysfunction in effector T cells [54]. T cell exhaustion is a dysfunctional state that occurs during chronic infections and cancer, characterized by reduced effector function, persistent expression of inhibitory receptors, and a unique transcriptional profile [55]. In the TME, exhausted T cells exhibit inhibitory receptor overexpression, reduced production of effector cytokines, and diminished cytolytic activity, resulting in the failure to eliminate cancer [56]. Clinically, the T cell exhaustion program safeguards CD8+ T cells from death due to overstimulation; thus, disrupting this program could potentially reduce the persistence of tumor-reactive T cells in patients with cancer [57]. IL-10 and TGF-β1 were secreted from CD4+CD25highFoxp3+ Tregs, which mediated immunosuppression in the HNSCC TME [58]. Currently, whether the presence of high Treg numbers affects the prognosis of patients with HNSCC is unclear. Controversial studies have shown that HNSCCs with high Treg frequencies have a poor prognosis, whereas others reported a better prognosis [59,60].

Various tumors have been associated with B cells that influence the prognosis of patients, either by promoting tumor progression or suppressing tumor growth [61]. PD-1 inhibition improves survival outcomes in patients with HNSCC and infiltrating B cells [62]. Single-cell analysis showed that patients had a better prognosis, greater immune cell infiltration, and distinct immune checkpoint levels, including elevated PD-1 levels, after B cell activation [63]. According to single-cell RNA sequencing analysis, TILs in patients with HPV-positive HNSCC include germinal center (GC), activated, and antibody-secreting B cell subsets. The anti-tumor immune response can also be inhibited by B and plasma cells in the TME, contributing to a better prognosis for patients. Furthermore, increased HPV-specific antibody titers are associated with an improved OS and reduced risk of recurrence in patients with HNSCC and HPV-positive infections [64,65]. Ruffin et al. reported that patients with HNSCC and HPV infections are characterized by the presence of tumor-infiltrating B cells and tertiary lymphoid structures (TLS) with GCs in their transcriptional signatures and spatial organization of immune cells in the tumor, both of which positively correlate with patient outcomes [66]. According to these findings, the phenotype and quantity of B cells may explain why patients with HNSCC and HPV infections receive a better prognosis [61]. Notably, HPV-positive tumors exhibit unique molecular characteristics and are generally associated with a more favorable prognosis compared to HPV-negative counterparts. This improved outcome is attributed to enhanced immunogenicity, better response to therapy, and a lower mutational burden, highlighting the significance of HPV status in both clinical decision-making and research investigations of the TME [67]. Table 1 summarizes the roles and clinical relevance of cellular components of the TME in HNSCC.

**Figure 2:**
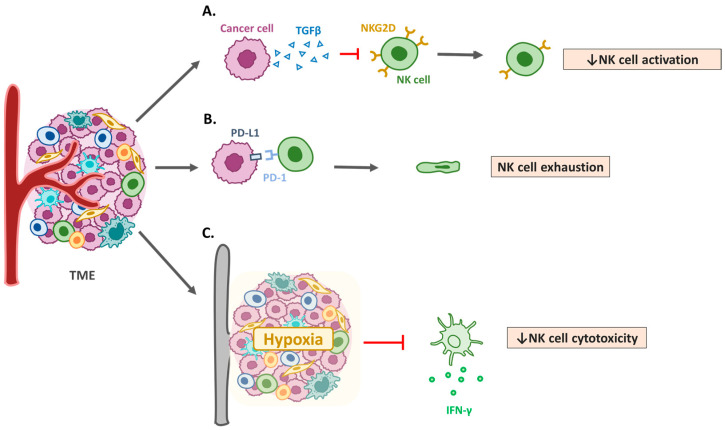
Mechanisms of natural killer cell dysfunction in HNSCC. (**A**) Transforming growth factor-β1 (TGF-β) and other immunosuppressive cytokines. Elevated levels of TGF-β in the tumor microenvironment (TME) suppress natural killer (NK) cell activation and reduce the expression of activating receptors, such as NK group 2D (NKG2D). (**B**) Programmed cell death protein 1 (PD-1)/PD-L1 axis and immune checkpoints. Expression of immune checkpoint molecules, such as PD-1, in NK cells and PD-L1 in tumor cells contributes to NK cell exhaustion in HNSCC. (**C**) Metabolic reprogramming and hypoxia. Hypoxic conditions and metabolic competition within the TME limit NK cell functionality, leading to reduced cytotoxicity.

**Figure 3:**
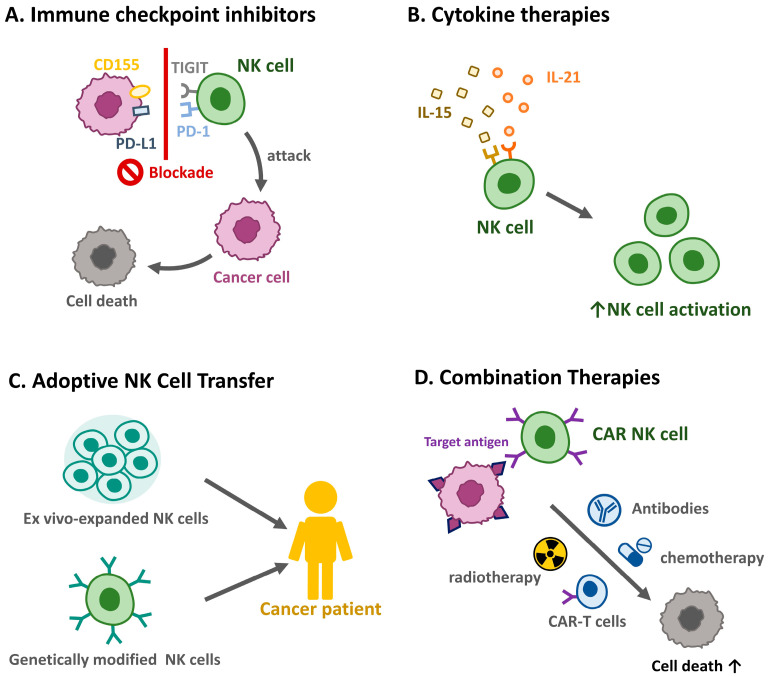
Therapeutic strategies targeting natural killer cells in HNSCC. Harnessing NK cells for therapeutic purposes offers promising avenues for improving HNSCC outcomes. (**A**) Immune checkpoint inhibitors. Blockade of inhibitory pathways, such as PD-1/PD-L1 and T cell immunoreceptor with immunoglobin and ITIM domains (TIGIT), enhances NK cell-mediated anti-tumor immunity. (**B**) Cytokine therapies. Administration of cytokines, such as IL-15 and IL-21, can boost NK cell proliferation and activation. (**C**) Adoptive NK cell transfer. The infusion of ex vivo-expanded or genetically modified NK cells is under investigation as a potential treatment modality. (**D**) Combination therapies. NK cell-based therapies combined with radiotherapy, chemotherapy, or other immunotherapies may synergistically enhance anti-tumor responses.

NK cells cooperate with other immune cells in the TME to destroy tumors and control metastases [68]. NK cells primarily exert their anti-tumor effects through direct cytotoxicity mediated by perforin and granzyme release, as well as through antibody-dependent cellular cytotoxicity [69]. Dysregulation of these mechanisms in HNSCC contributes to immune evasion [70]. In addition, NK cells interact with dendritic cells, macrophages, and T cells in the TME, thereby influencing the overall immune response [71]. NK-derived cytokines, such as interferon (IFN)-γ, can also modulate T cell activation and dendritic cell maturation [72]. The HNSCC TME promotes immune evasion by impairing NK cell recruitment, survival, and cytotoxicity (Figure 2). Notably, treatment with IL-15 in mice bearing HNSCC tumors induces the differentiation of NK cells into CD49a+ cells, which produce higher amounts of IFN-γ to suppress tumor growth [73]. Mandal et al. demonstrated that most immune-infiltrated HNSCC tumors had the highest median Treg/CD8+ T cell ratio and CD56dim NK cell infiltration. Patients with HNSCC and CD8+ T and CD56dim NK cell infiltrations have superior survival rates [46]. In the mouse OSCC MOC2 cell line, peripheral CXCR2+ neutrophilic-MDSCs pathologically accumulate and suppress NK cell function through the translocation and release of hydrogen peroxide (H_2_O_2_). Murine NK cells adoptively transferred into tumors were more effective in infiltrating, activating, and attacking tumors after MDSC trafficking was inhibited by orally bioavailable SX-682 [74]. Chi et al. established a data mining model to respond to more effective immunotherapy in low-risk patients with HNSCC [75]. Together, these findings emphasize the importance of NK cells in tumor control and the potential of targeted strategies to enhance their anti-tumor effects (Figure 3).

### 2.2. Endothelial Cells and Angiogenesis

Angiogenesis, the process of new blood vessel formation from the preexisting vasculature, is a hallmark of cancer progression and plays a critical role in the growth and metastasis of HNSCC [76]. Endothelial cells in the TME contribute to the formation of an abnormal and leaky vasculature, which facilitates tumor cell dissemination and creates regions of hypoxia [77]. Hypoxia induces the expression of hypoxia-inducible factors (HIFs), which promote angiogenesis, metabolic reprogramming, and resistance to therapy [78]. In HNSCC, endothelial cells are activated by pro-angiogenic factors, primarily vascular endothelial growth factor (VEGF) [79]. VEGF signaling induces endothelial cell proliferation, migration, and tube formation, promoting the development of a disorganized and leaky vasculature that supports tumor growth and facilitates metastasis [80]. Other angiogenic mediators, such as fibroblast growth factors (FGFs) and angiopoietins (ANGs), also contribute to endothelial cell activation and angiogenesis [81]. Endothelial cells in the HNSCC TME contribute to immune suppression by expressing immune checkpoint molecules, such as PD-L1 and Fas ligand (FasL), which inhibit T cell activation and induce the apoptosis of cytotoxic T cells [82]. Tumor-infiltrating immune cells exacerbate tumor-associated endothelial cell dysfunction by secreting pro-angiogenic factors, further restricting immune infiltration [83]. Additionally, endothelial cells can limit immune cell infiltration into the tumor by downregulating the expression of adhesion molecules necessary for immune cell trafficking [84]. Therefore, understanding the intricate interactions between endothelial and immune cells is critical for developing combined therapies that normalize tumor vasculature and enhance anti-tumor immunity.

Hypoxia within the tumor mass acts as a potent driver of angiogenesis by stabilizing HIF-1α, which upregulates VEGF expression [85]. The hypoxic environment not only stimulates endothelial cells but also alters their interactions with cancer and immune cells, further enhancing angiogenesis and immune evasion [86]. Given the central role that angiogenesis plays in HNSCC progression, targeting endothelial cells and angiogenic pathways represents a promising therapeutic approach [87]. Anti-angiogenic agents, such as VEGF (e.g., bevacizumab) and multi-tyrosine kinase (e.g., sorafenib) inhibitors, have been investigated for their ability to normalize the tumor vasculature, enhance drug delivery, and improve the efficacy of immunotherapy [88]. Although anti-angiogenic therapies show potential, their efficacy in HNSCC is limited by resistance mechanisms and off-target effects [89]. Combination therapies that integrate angiogenesis inhibitors with immune checkpoint inhibitors or conventional treatments, such as radiotherapy, are being explored to overcome these challenges [90]. Notably, several PD-1/PD-L1 inhibitors, including pembrolizumab, nivolumab, or atezolizumab, are now approved for the treatment of recurrent or metastatic HNSCC and are being actively studied in combination with anti-angiogenic agents. Therefore, understanding the crosstalk among endothelial, cancer, and immune cells in the HNSCC TME is critical for designing more effective strategies. Table 2 summarizes the roles of endothelial cells and angiogenesis in the HNSCC TME.

### 2.3. ECM

The ECM is a dynamic and complex network of proteins, glycoproteins, and proteoglycans that provides structural support to tissues and regulates numerous cellular functions, including proliferation, migration, and differentiation [92]. In addition, the ECM in tumors differs markedly from that in normal tissue in terms of abundance, composition, organization, and mechanical properties. In HNSCC, interactions between malignant epithelial and stromal cells drive the upregulation of specific ECM components, which facilitate carcinoma cell migration, alter the cytokine environment, and enhance immune evasion [93]. A previous article summarized that VEGF, FGF-2, and ANG-2 activate protein kinase B (AKT) and mitogen-activated protein kinase signaling pathways, which drive both the EMT and vessel formation in OSCC and oral potentially malignant disorders [94]. ECM remodeling is mediated by matrix metalloproteinases (MMPs) and other proteolytic enzymes, which degrade ECM components and release growth factors that promote tumor invasion and metastasis in HNSCC [1,95]. MMP-2 secreted by senescent CAFs-conditioned medium promoted keratinocyte dis-cohesion and facilitated epithelial invasion into collagen gels through a TGF-β-dependent mechanism in OSCC [96]. CAFs are the principal source of ECM components and remodeling enzymes in the HNSCC TME. CAFs not only secrete collagens, fibronectin, and proteases such as MMPs to remodel the ECM, but also interact with cancer and immune cells to promote tumor progression, angiogenesis, and immune suppression. Numerous studies have shown that a high abundance of CAFs in tumor tissues is associated with increased tumor invasiveness, therapeutic resistance, and poor clinical prognosis in patients with HNSCC [97,98,99]. Therefore, targeting CAFs and their interactions with the ECM represents a promising strategy to improve patient outcomes. The ECM of the HNSCC TME is characterized by an altered composition, including elevated levels of collagen, fibronectin, laminin, and hyaluronic acid [26,100]. These changes promote a pro-tumorigenic environment by influencing cancer cell behavior, facilitating immune evasion, and enhancing angiogenesis [101]. Excessive collagen deposition and crosslinking stiffen the ECM, promoting tumor cell invasion and metastasis by enhancing mechanotransduction signaling pathways [102]. Additionally, these glycoproteins provide binding sites for integrins on cancer cells, activating pathways that drive migration, survival, and the EMT [103]. Elevated levels of hyaluronic acid contribute to ECM hydration and create a physical barrier that impedes immune cell infiltration [104]. These findings underscore the pivotal role that ECM dynamics play in HNSCC progression and highlight the potential therapeutic targets for disrupting tumor-promoting ECM modifications.

### 2.4. Immune Evasion Mechanisms and Immune Checkpoints

HNSCC employs various strategies to evade the immune system, many of which are facilitated by the TME [105]. These mechanisms include the expression of immune checkpoint molecules, secretion of immunosuppressive cytokines, and alteration of antigen presentation pathways [91]. Recent single-cell transcriptomic analyses have highlighted TGF-β signaling as a key regulator of functional interactions between CAFs and a specific subset of mesenchymal cancer cells [106]. Immune checkpoint molecules, such as PD-1 and its ligand, PD-L1, play a critical role in maintaining immune homeostasis by preventing autoimmunity [107]. However, tumor cells can exploit these pathways to evade immune surveillance. In HNSCC, PD-L1 is often overexpressed in tumor and immune cells within the TME, leading to the inhibition of T cell activation and function [108]. Tregs, known for their immunosuppressive properties, play a crucial role in OSCC progression and patient prognosis [109]. Their function within the OSCC TME is driven by metabolic reprogramming, involving key pathways that include the tryptophan–kynurenine–aryl hydrocarbon receptor, phosphatidylinositol 3-kinase (PI3K)-AKT-mechanistic target of rapamycin (mTOR), and nucleotide metabolism to enhance their suppressive activity [108,110,111,112]. A recent study revealed significantly higher B and T-lymphocyte attenuator (BTLA) expression in OSCC, along with increased levels of PD1, PD-L1/2, and CD96. Moreover, strong correlations between BTLA and other immune checkpoints suggest that it plays a role in OSCC immune evasion [113]. Immune checkpoints play critical roles in immune evasion in HNSCC. The key pathways involved in this process include PD-1/PD-L1, CTLA-4, T cell immunoglobulin mucin 3 (TIM-3), lymphocyte activation gene 3 (LAG-3), and TIGIT [114]. TIM-3 expression alone is not sufficient to induce TIL exhaustion; instead, its coexpression with PD-1 is required for significant TIL dysfunction [115]. High PD-1 levels in both TIM-3-negative and TIM-3-positive TILs are linked to the increased expression of B-lymphocyte-induced maturation protein-1 and basic leucine zipper ATF-like transcription factor, which are transcription factors that suppress T cell proliferation and cytokine production [116]. In patients with HNSCC, the elevated expression of TIM-3, an immune checkpoint receptor that promotes cell proliferation through the AKT/S6 pathway, serves as a marker of exhausted TILs [117]. Deng et al. reported that LAG-3 is upregulated in CD4+ and CD8+ T cells and Tregs within the TME. High LAG-3 expression correlates with a poorer prognosis in patients with HNSCC. In vivo experiments demonstrated that blocking LAG-3 with specific antibodies retards tumor growth by enhancing CD8+ T cell-mediated anti-tumor responses and reducing immunosuppressive cell populations [118]. TIGIT overexpression in CD8+ and CD4+ T cells is correlated with HNSCC progression and a poor treatment response, and TIGIT/PD-1/LAG-3 axis activation is correlated with tumor progression and the development of an immunosuppressive microenvironment [119]. Overall, understanding the intricate network of immune checkpoints in HNSCC provides valuable insights for the development of targeted immunotherapies aimed at restoring anti-tumor immunity and improving patient outcomes (Table 3).

The TME plays a central role in promoting immune evasion in HNSCC through multiple mechanisms that collectively suppress antitumor immunity and facilitate tumor progression [120]. These include upregulation of immune checkpoint molecules such as PD-L1, recruitment of immunosuppressive cell populations including Tregs, MDSCs, and TAMs, secretion of immunosuppressive cytokines such as IL-10, TGF-β, induction of T cell exhaustion, and metabolic alterations that hinder effector immune cell function [121]. To enhance clarity and translational relevance, these mechanisms can be summarized into four major categories: (1) checkpoint molecule overexpression including PD-L1/PD-1 axis, (2) immuno-suppressive cellular infiltration, (3) cytokine-mediated immune suppression, and (4) metabolic and oxidative stress-induced immune dysfunction.

Importantly, PD-L1 overexpression has emerged as a critical feature of immune escape in HNSCC, with implications beyond its predictive value for immunotherapy [122]. Elevated PD-L1 expression is also associated with poor prognosis, increased tumor aggressiveness, and resistance to both chemoradiotherpy and targeted agents [123]. These insights underscore the potential of PD-L1 not only as a biomarker for therapeutic selection but also as a target for prognostic stratification.

From a translational perspective, several therapeutic strategies are being actively investigated to reverse the immunosuppressive TME and restore effective antitumor immunity. These include immune checkpoint inhibitors including anti-PD-1/PD-L1 and anti-CTLA-4 antibodies, TME-targeting agents such as CSF1R inhibitors or indoleamine-pyrrole 2,3-dioxygenase 1 (IDO1) inhibitors, and combinatorial approaches that integrate immunotherapy with radiation, chemotherapy, or targeted therapies [124]. The development of novel agents that modulate the TME, along with biomarkers to guide their use, holds promise for improving outcomes in HNSCC and overcoming current therapeutic resistance. These strategies highlight the critical need to translate our growing under-standing of TME biology into effective clinical interventions.

### 2.5. Immune Evasion and the Impact of TP53 Mutations in HNSCC

Effective anti-tumor immunity relies on the recognition of tumor antigens by T cells [125]. However, HNSCC cells can downregulate the expression of MHC molecules and antigen-processing machinery (APM) components, thereby impairing antigen presentation and preventing immune detection [126]. Additionally, the loss of heterozygosity (LOH) of the human leukocyte antigen (HLA) locus is a common feature of HNSCC, which further contributes to the evasion of immune surveillance [127] and correlates with a poor prognosis, serving as a potential prognostic marker [128].

*TP53*, a crucial tumor suppressor gene, plays a central role in maintaining genomic stability by regulating cell cycle arrest, DNA repair, and apoptosis [129]. In HNSCC, *TP53* mutations are among the most common genetic alterations often associated with tumor progression, resistance to therapy, and poor clinical outcomes [130]. The tumor suppressor *TP53* plays a central role in maintaining genomic stability and regulating anti-tumor immunity. Wild-type *TP53* supports immune surveillance by promoting antigen presentation through MHC class I expression, suppressing immunosuppressive cytokines such as TGF-β and IL-10, and downregulating PD-L1 expression via modulation of the Janus kinase/STAT and nuclear factor kappa-light-chain-enhancer of activated B cells (NF-κB) signaling pathways [126,131,132]. In contrast, mutation of *TP53* impairs these immune-regulatory functions and contributes to an immunosuppressive TME. Mechanistically, *TP53* mutations are associated with reduced expression of MHC class I molecules, defective antigen processing, increased infiltration of Tregs and MDSCs, and diminished cytotoxic T cell activity [126]. These alterations decrease tumor immunogenicity and lead to a poor response to PD-1/PD-L1 immune checkpoint blockade. Disruptive *TP53* mutations, in particular, are correlated with lower immune infiltration and reduced responsiveness to anti–PD-1 therapy in HNSCC [131,133]. These mutations can be broadly categorized as disruptive or nondisruptive, with disruptive mutations leading to the complete loss of p53 function [134]. Shi et al. established a risk score model based on *TP53* mutation-associated genes to assess the prognosis and therapeutic responses in patients with HNSCC. They found that *TP53* mutations, the most common in HNSCC, correlated with suppressed immune signatures and a poorer OS. Patients with higher risk scores exhibited reduced responses to anti-PD-1 immunotherapy but increased sensitivity to certain chemotherapies [133]. In contrast, the findings revealed that patients with disruptive *TP53* mutations experienced higher rates of locoregional recurrence and a lower OS than those with nondisruptive or no mutations. Mechanistically, disruptive *TP53* mutations are associated with the failure to undergo radiation-induced senescence, leading to increased tumor cell proliferation post-treatment [135]. Previous studies have also shown that patients with disruptive *TP53* mutations exhibit higher rates of locoregional recurrence and a reduced OS, particularly in response to radiation therapy [136,137]. Caponio et al. analyzed the association between high-risk *TP53* mutations and primary treatment responses and found that such mutations were linked to poorer outcomes. The study also evaluated the effectiveness of two classification systems for *TP53* mutations and provided insights into their potential clinical utility [131]. In conclusion, the ability of HNSCC to evade immune detection is driven by multiple mechanisms, including the downregulation of MHC molecules, alterations in the APM, and HLA LOH, all of which contribute to a poor prognosis. Additionally, *TP53* mutations, particularly disruptive ones, play a significant role in tumor progression, treatment resistance, and immune suppression. These mutations are associated with high rates of locoregional recurrence, reduced OS, and impaired responses to radiation and immunotherapy. Given these findings, assessing *TP53* mutation status and HLA LOH could serve as valuable prognostic markers for guiding personalized treatment strategies and improving therapeutic outcomes in patients with HNSCC.

## 3. Conclusions

The TME plays a pivotal role in the immune evasion of HNSCC, contributing to tumor progression and resistance to therapy. Advances in our understanding of the molecular and cellular mechanisms underlying this process have opened new avenues for therapeutic interventions. Targeting the TME and its associated immunosuppressive pathways may enhance anti-tumor immunity and improve the prognosis of patients with HNSCC. Further research is needed to identify effective combination strategies and biomarkers to optimize the use of these therapies in clinical practice.

## Figures and Tables

**Table 1:** Cellular components of the TME in HNSCC: roles and clinical relevance.

Cell Type	Function in TME	Clinical Implications	References
CAFs	Secrete cytokines, growth factors, and ECM components; promote tumor growth, angiogenesis, and immune modulation	Facilitate tumor invasion and immune cell recruitment; contribute to immunosuppressive TME	[29,40,41]
CD8+ Cytotoxic T Cells	Mediate anti-tumor immunity through cytolysis and cytokine secretion	Often functionally exhausted in HNSCC, impairing tumor clearance	[47,55,56,57]
Tregs	Suppress effector T cells via IL-10 and TGF-β1; modulate immune tolerance	Conflicting prognostic value in HNSCC; may promote or inhibit tumor progression	[58,59,60]
MDSCs	Suppress T cell activation and contribute to immunosuppression	Accumulate in HPV-negative tumors; linked to immune evasion	[42,48]
TAMs	Often exhibit M2-like (CD163+) phenotype; secrete anti-inflammatory cytokines and support tumor growth	High CD163+ TAMs correlate with poor prognosis, reduced OS and PFS	[49,50,51,52]
Exhausted T Cells	Exhibit reduced cytokine production and overexpression of inhibitory receptors (PD-1, TIGIT)	Impaired anti-tumor immunity; potential targets for immunotherapy	[54,55,56,57]
B Cells and Plasma Cells	Can produce antibodies, present antigens, or suppress T cell activity; phenotypes include GCBs, ABCs, and PCs	Dual roles: associated with better prognosis, especially in HPV+ tumors; PD-1 expression on B cells may predict ICI response	[61,62,63,64,65,66]
TILs	Comprise CD8+, CD4+, B cells; enriched in HPV+ HNSCC	High TILs associated with improved survival; CD4+/D8+ ratios and B cell phenotype correlate with recurrence-free survival	[43,44,53,64,65,66]
HPV Status Influence	HPV+ tumors exhibit lymphocyte-rich immune microenvironment; HPV−tumors have more suppressive myeloid cells	Better clinical outcomes in HPV+ patients; B cell- and TLS-related transcriptional signatures positively correlate with prognosis	[42,43,44,45,46,64,65,66]

**Table 2:** Role of endothelial cells and angiogenesis in HNSCC TME.

Category	Description	Key Factors/Examples	References
Function of angiogenesis	Formation of new blood vessels to supply nutrients and oxygen; facilitates tumor growth and metastasis	-	[76]
endothelial cell Activation	Stimulated by pro-angiogenic signals leading to proliferation, migration, and tube formation	VEGF, FGFs, and ANGs	[79,80,81]
Hypoxia-induced response	Hypoxia stabilizes HIF-1α, upregulating VEGF and enhancing angiogenesis and metabolic adaptation	HIF-1α and VEGF	[78,85]
Vascular abnormalities	Tumor vasculature is disorganized and leaky, contributing to poor perfusion, hypoxia, and therapy resistance	Tumor endothelial cells	[77,80]
Immune suppression by endothelial cells	Endothelial cells express checkpoint molecules to inhibit T cell activation and reduce immune cell infiltration	PD-L1 and FasL	[82,91]
Immune cell interaction	Tumor-infiltrating immune cells secrete pro-angiogenic factors; endothelial cells downregulate adhesion molecules, restricting immune trafficking	Pro-angiogenic cytokines, ↓ intercellular adhesion molecule-1/vascular cell adhesion molecule-1,	[83,84]
Therapeutic targets	Targeting angiogenesis and immune checkpoints to normalize vasculature, restore immune infiltration, and enhance therapy delivery	VEGF inhibitors (bevacizumab), tyrosine kinase inhibitors (sorafenib), and anti-PD-1/PD-L1 (pembrolizumab and nivolumab)	[21,22,87,88]
Limitations of therapy	Resistance mechanisms and off-target effects reduce the efficacy of anti-angiogenic monotherapy	-	[89,90]
Emerging/Current strategies	Combining anti-angiogenic agents with immune checkpoint inhibitors or radiotherapy to overcome resistance and restore immune infiltration	Anti-VEGF + anti-PD-1/PD-L1 (atezolizumab) or radiotherapy	[90,91]

**Table 3:** Immune evasion mechanisms in HNSCC via checkpoint molecules and TME modulation.

Immune Checkpoint/Pathway	Cellular/Molecular Source	Mechanism of Immune Evasion	Clinical Relevance/Impact	Available Drug(s)	References
TGF-β Signaling	CAFs and mesenchymal-like cancer cells	Promotes immunosuppression and modulates CAF–tumor interactions	Identified as key in single-cell transcriptomics in HNSCC TME	Galunisertib (LY2157299)	[106]
PD-1/PD-L1	Tumor cells, TILs, and myeloid cells	Inhibits T cell activation and cytokine production; promotes T cell exhaustion	Overexpressed in HNSCC; predictor for response to anti-PD-1 therapy	Nivolumab, Pembrolizumab, Atezolizumab, Durvalumab	[107,108]
Tregs	CD4+CD25+FoxP3+ cells	Suppress effector T cell functions via cytokines (IL-10 and TGF-β); metabolic reprogramming enhances suppressive phenotype	Enriched in OSCC TME; associated with poor prognosis	Indirect target via anti-CTLA-4 (Ipilimumab)	[109,110,111,112]
Metabolic checkpoints (Kynurenine–aryl hydrocarbon receptor, PI3K–mTOR, and nucleotide metabolism)	Tregs and tumor cells	Metabolic adaptation supports Treg function, inhibits effector T cell proliferation and survival	Enhances suppressive TME and promotes immune tolerance in OSCC	Indoximod, Epacadostat (IDO1 inhibitors; in trials)	[110,111,112]
BTLA (B and T lymphocyte attenuator)	T and B cells	Negatively regulates lymphocyte activation	Correlated with PD-1, PD-L1/2, CD96 in OSCC; emerging marker of immune evasion	-	[113]
CD96	NK cells and T cells	Negatively regulates NK cell-mediated cytotoxicity	Increased expression in OSCC; role in immune escape mechanism	-	[113]
CTLA-4	Tregs and activated T cells	Inhibits T cell priming by outcompeting CD28 for B7 ligands	Contributes to immune suppression and Treg function in TME	Ipilimumab, Tremelimumab	[114]
TIM-3	Exhausted CD8+ T cells and Tregs	Requires co-expression with PD-1 to fully induce TIL exhaustion; activates AKT/S6 signaling	Marker of dysfunctional TILs in HNSCC	MBG453 (Sabatolimab), TSR-022 (clinical trials)	[115,116,117]
LAG-3	CD4+, CD8+ T cells, and Tregs	Suppresses T cell effector function; inhibits proliferation and cytokine production	High expression linked to poor prognosis; blockade restores CD8+ T cell function	Relatlimab (approved with Nivolumab)	[118]
TIGIT	CD4+ and CD8+ T cells	Suppresses NK and T cell activity; promotes Treg function	Upregulated in progressive HNSCC; co-expressed with PD-1 and LAG-3 in immunosuppressive axis	Tiragolumab, Domvanalimab (in clinical trials)	[119]

## Data Availability

Not applicable.

## Abbreviations

The following abbreviations are used in this manuscript:

AKT	Protein kinase B
ANG	Angiopoietin
APM	Antigen-processing machinery
BTLA	B and T-lymphocyte attenuator
CAF	Cancer-associated fibroblast
CD	Cluster of differentiation
CTLA-4	Cytotoxic T-lymphocyte-associated protein 4
CXCL	C-X-C motif chemokine ligand
CXCR	C-X-C chemokine receptor
ECM	Extracellular matrix
EMT	Epithelial–mesenchymal transition
FasL	Fas ligand
FGF	Fibroblast growth factor
FOXP3	Forkhead box protein P3
GC	Germinal center
HIF	Hypoxia-inducible factor
HLA	Human leukocyte antigen
HNSCC	Head and neck squamous cell carcinoma
HPV	Human papillomavirus
IDO1	Indoleamine-pyrrole 2,3-dioxygenase
IFN	Interferon
IHC	Immunohistochemistry
IL	Interleukin
LAG-3	Lymphocyte activation gene 3
LOH	Loss of heterozygosity
MDSC	Myeloid-derived suppressor cells
MHC	Major histocompatibility complex
MMP	Matrix metalloproteinase
mTOR	Mechanistic target of rapamycin
NF-κB	Nuclear factor kappa-light-chain-enhancer of activated B cells
NK	Natural killer
OS	Overall survival
OSCC	Oral squamous cell carcinoma
PD-1	Programmed cell death protein 1
PD-L1	Programmed death-ligand 1
PFS	Progression-Free Survival
PI3K	Phosphatidylinositol 3-kinase
STAT	Signal transducer and activator of transcription
TAM	Tumor-associated macrophage
TGF	Transforming growth factor
TIGIT	T cell immunoreceptor with immunoglobin and ITIM domains
TIL	Tumor-infiltrating lymphocyte
TIM-3	T cell immunoglobulin mucin 3
TLS	tertiary lymphoid structure
TME	Tumor microenvironment
Treg	Regulatory T cell
VEGF	Vascular endothelial growth factor
